# Influence of Y Doping on WO_3_ Membranes Applied in Electrolyte-Insulator-Semiconductor Structures

**DOI:** 10.3390/membranes12030328

**Published:** 2022-03-15

**Authors:** Chyuan-Haur Kao, Yu-Ching Liao, Chi-Chih Chuang, Yi-Hsuan Huang, Chang-Hsueh Lee, Shih-Ming Chen, Ming-Ling Lee, Hsiang Chen

**Affiliations:** 1 Department of Electronic Engineering, Chang Gung University, 259 Wen-Hwa 1st Road, Kwei-Shan District, Taoyuan City 333, Taiwan; chkao@mail.cgu.edu.tw (C.-H.K.); chkao@mail.cgu.edu (Y.-C.L.); 2 Kidney Research Center, Department of Nephrology, Chang Gung Memorial Hospital, Chang Gung University, No. 5 Fuxing St., Guishan District, Taoyuan City 333, Taiwan; 3 Department of Electronic Engineering, Ming Chi University of Technology, 284 Gungjuan Rd., Taishan District, New Taipei City 243, Taiwan; 4 Department of Applied Materials and Optoelectronic Engineering, National Chi Nan University, Puli Nantou 545, Taiwan; s109328011@mail1.ncnu.edu.tw (C.-C.C.); s109328038@mail1.ncnu.edu.tw (Y.-H.H.); s110328506@mail1.ncnu.edu.tw (C.-H.L.); s107328009@mail1.ncnu.edu.tw (S.-M.C.); 5 Department of Electro-Optical Engineering, Minghsin University of Science and Technology, No. 1, Xinxing Rd., Xinfeng, Hsinchu 304, Taiwan

**Keywords:** tungsten trioxide, yttrium doping, annealing, electrolyte-insulator-semiconductor, membranes, defects

## Abstract

In this paper, tungsten oxide (WO_3_) is deposited on a silicon substrate applied in electrolyte-insulator-semiconductor structures for pH sensing devices. To boost the sensing performance, yttrium (Y) is doped into WO_3_ membranes, and annealing is incorporated in the fabrication process. To investigate the effects of Y doping and annealing, multiple material characterizations including X-ray diffraction (XRD), X-ray photoelectron spectroscopy (XPS), atom force microscopy (AFM), scanning electron microscopy (SEM), and transmission electron microscopy (TEM) are performed. Material analysis results indicate that annealing and Y doping can increase crystallinity, suppress defects, and enhance grainization, thereby strengthening membrane sensing capabilities in terms of sensitivity, linearity, and reliability. Because of their stable response, high reliability, and compact size, Y-doped WO_3_ membranes are promising for future biomedical applications.

## 1. Introduction

After the invention of the ion-sensitive field-effect transistor ISFET in 1970 [1], novel materials and treatments have been demonstrated with the development of FET sensing devices [2]. Among these, electrolyte-insulator-semiconductor (EIS) sensors [3] have received attention for use in various biological detection such as ion sensing, DNA detection, and antibody assessment. However, traditional clinical pH-sensing measurements require more time and money to analyze than EIS sensors in blood or human secretion samples, and long-term, continuous measurements are unreliable. Therefore, stable and reliable semiconductor-based pH sensors can benefit patients in rapid, simple, and inexpensive testing [4,5,6]. To further improve sensing device performance, various materials have been utilized as sensing membrane insulation. Due to the low capacitance and inferior electric field modulation in SiO_2_ binary oxides such as Ta_2_O_5_ [7], La_2_O_3_ [8], and ZrO_2_ [9] have emerged as replacements for traditional SiO_2_. Recently, WO_3_ [10] and Y_2_O_3_ [11] have been demonstrated as membrane materials. Yttrium oxides exhibit a high dielectric constant of 14–18, a large conduction band offsets of 2.3 eV, a wide energy bandgap of 5.6 eV, and excellent dielectric constants. [8] However, Y doping, which may form Y_2_O_3_ oxide in membrane oxides and boost sensing performance, has not been reported [12]. In this study, WO_3_ and Y are co-sputtered on a substrate to improve the sensing behaviors and material properties [13,14]. RTA annealing at various temperatures is performed on the membrane oxides [15]. The influence of Y doping on the material has been discussed in many studies. According to a report by Liu et al. [16], doping with a small amount of Y^3+^ significantly increases conductivity, [17]. As the amount of doping increases, the conductivity first increases and then decreases, indicating that a small amount of Y^3+^ doping can increase the size of the grains. According to a report by Wen et al. [18], it is found that Y doping helps to improve the surface, structure, optical, and electrical properties of ZnO because the ion radius of Zn (0.740 Å) is smaller than that of Y (0.890 Å) [19,20]. The results show that the structure is relatively stable and increases with Y content. A report by William Lee et al. [21] has proved that Y^3+^ replaces Ce and controls the formation of oxygen vacancies [22]. However, Y doping for improvement of EIS sensing membrane behaviors has not been clearly reported [23]. In this study, yttrium is doped in WO_3_ membranes, and WO_3_ and Y-doped WO_3_ sensing films are compared using multiple material characterizations techniques and pH-sensing measurements [24]. Results indicate that Y doping combined with annealing can significantly improve sensing behaviors. Y-doped WO_3_ membranes in EIS structures are promising for future biomedical applications.

## 2. Materials and Methods

**Figure 1:**
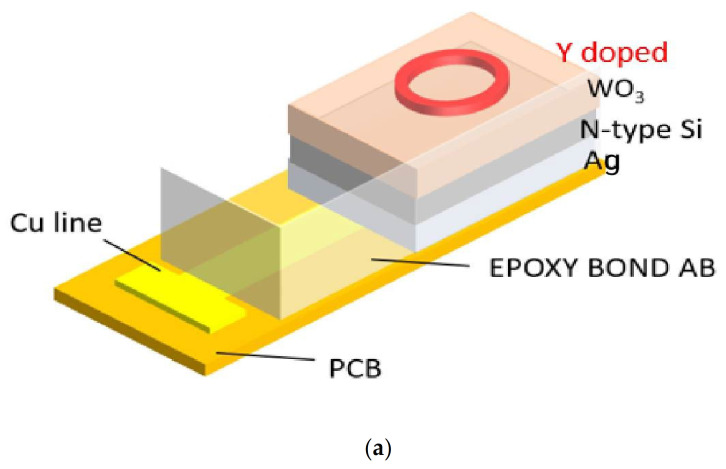

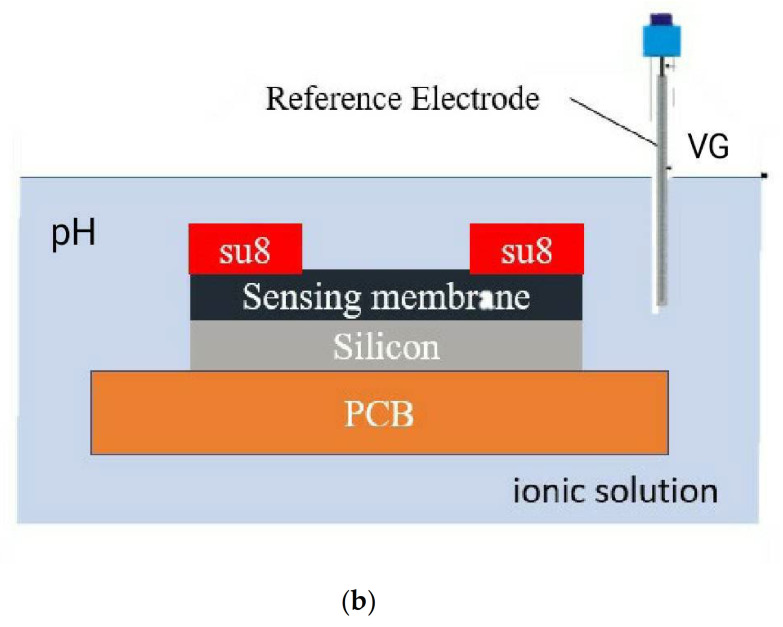
(**a**) The Y-doped WO_3_ of EIS structure (**b**) The device under operation.

The following are the fabrication processes of the EIS sensor with the WO_3_ sensing film and Y-doped WO_3_ sensing film. The WO_3_ sensing membrane is deposited on silicon substrate by RF sputtering with RF power of 100 W. The chamber pressure is 10 mTorr and the deposition gas ratio is Ar:O_2_ = 20:5. The deposition thickness of WO_3_ is 60 nm. The Y-doped WO_3_ sensing membrane is co-sputtered on silicon substrate by RF Sputter with WO_3_ target and Y target, in which the two RF powers are 100 and 120 W, respectively. The deposited pressure is 10 mTorr, the deposition gas ratio is Ar:O_2_ = 20:5, and the deposition thickness is 65 nm. The WO_3_ sensing film and the Y-doped WO_3_ sensing film are given RTA treatment. The annealing temperatures are 400, 500, and 600 °C, respectively; the annealing time is 30 s in an oxygen environment. Then, adhesive silicone gel is used to define the sensing window and, conductive silver glue (Ag) is then used to fix it onto a PCB board. Finally, AB glue is used for packaging to prevent oxidation. The detailed EIS structure of the Y-doped WO_3_ sensing membrane is illustrated in Figure 1a. The device under operation is shown in Figure 1b.

## 3. Results and Discussion

**Figure 2:**
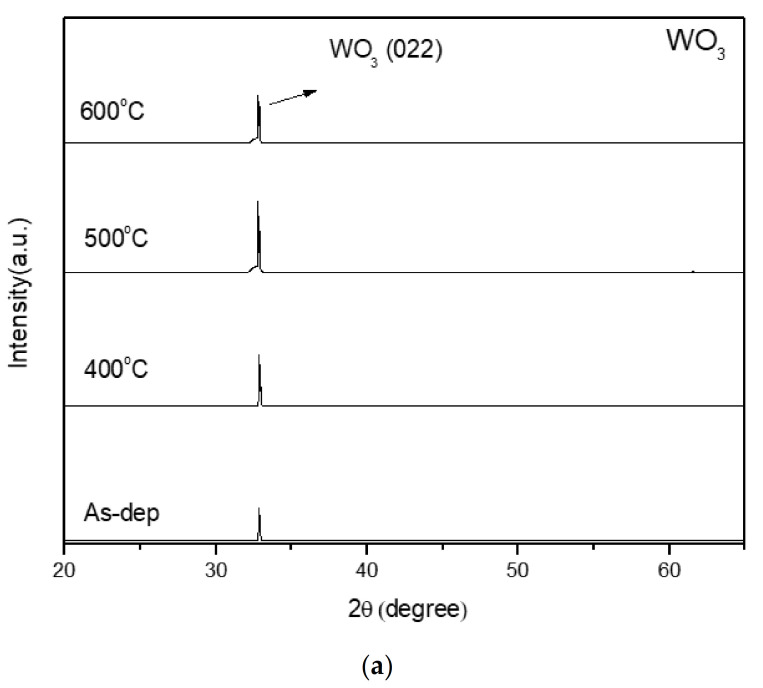

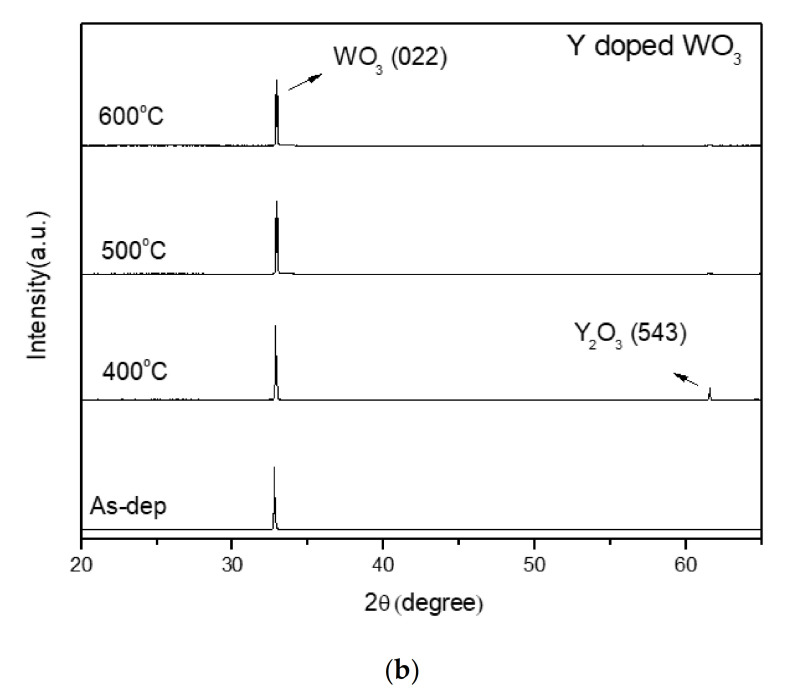
(**a**) XRD of the WO_3_ film after annealing at various temperatures in O_2_ ambient for 30 s. (**b**) XRD of the Y-doped WO_3_ film after annealing at different temperatures in O_2_ ambient for 30 s.

Figure 2a,b shows the XRD analysis of the WO_3_ sensing membrane and the Y-doped WO_3_ sensing film without annealing and annealing at 400, 500, and 600 °C, respectively. The two types of samples have the same diffraction peak (022), which can be observed at the 2-Theta value of 32.9, and the peak is attributed to monoclinic WO_3_ (022). In the Y-doped WO_3_ sensing film, there are two diffraction peaks (022) and (543) [25,26], which can be observed at the 2-Theta value of 32.9 and 61.6. The peak at the 2-Theta value of 61.6 is attributed to Y_2_O_3_ (543). After annealing, the peak of WO_3_ (022) increases appreciably. Of the samples, the one with annealing at 400 °C has the strongest peak intensity, and the peak of Y_2_O_3_ (543) shows better crystallization. The XRD comparison of the WO_3_ sensing membrane and the Y-doped WO_3_ sensing membrane shows that the peak of WO_3_ (022) is stronger after doping.

Using XRD analysis, our study shows that the WO_3_ sensing film annealed at 500 °C has the best crystallization with a high intensity of the peak WO_3_ (022). On the other hand, the Y-doped WO_3_ sensing film annealed at 400 °C has two peaks of WO_3_ (022) and Y_2_O_3_ (543), showing a small amount of Y^3+^ can enhance crystallization.

**Figure 3:**
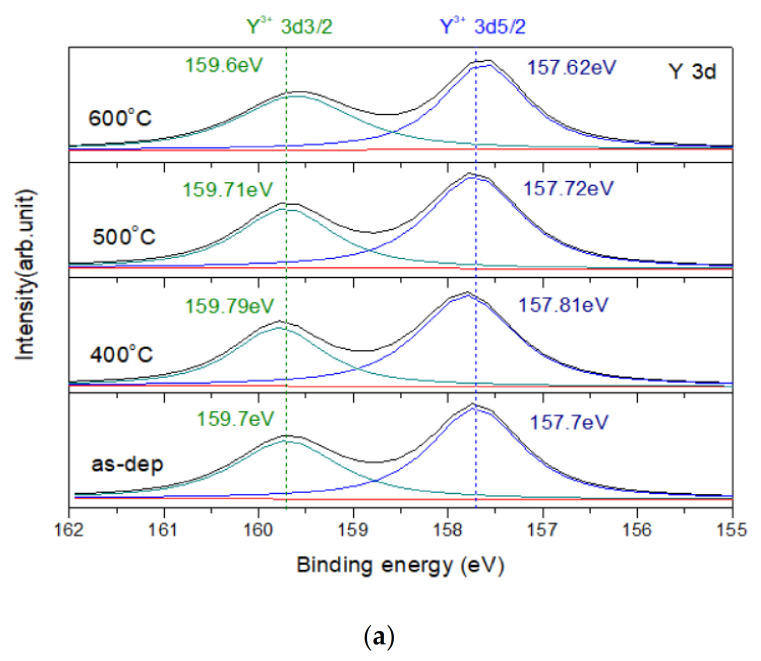

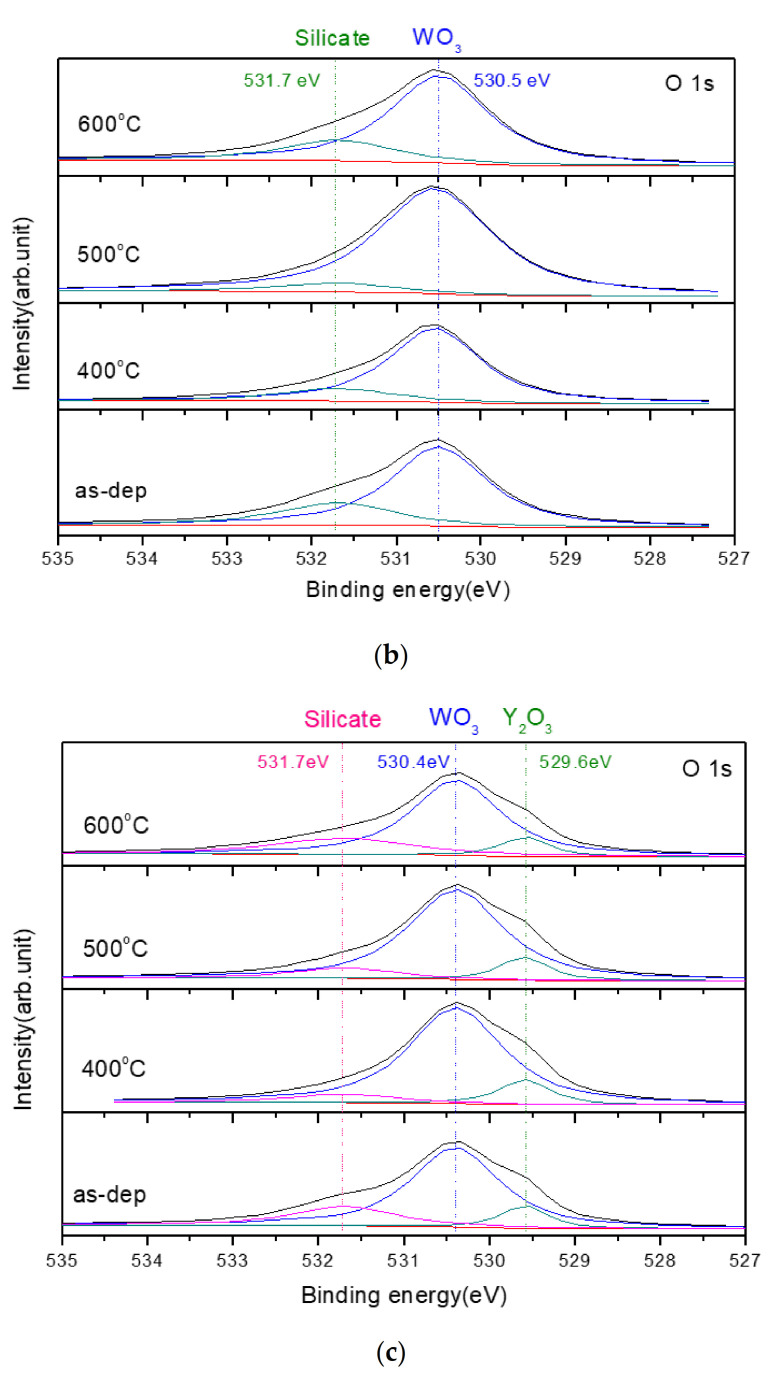
(**a**) The Y 3D XPS spectra of Y-doped WO_3_ film annealed at different temperatures in O_2_ ambient for 30 s. (**b**) O 1s of WO_3_ film. (**c**) O 1s of Y-doped WO_3_, annealed at various temperatures in O_2_ ambient for 30 s.

The Y 3D XPS spectra of Y-doped WO_3_ are shown in Figure 3a. There are two peaks located at the binding energy values of 157.7 [27] and 159.7 eV [28] for Y^3+^ in the case of as-dep, respectively. It can be observed that the binding energy is higher than the standard binding energy located at the values of 156.6 and 157.4 eV. The higher binding energy means the Y ions have been doped in the WO_3_ sensing film. Based on the literature [29,30,31,32], Y–O bonds are stronger than W–O bonds.

Figure 3b,c show the O 1s XPS spectra of the WO_3_ membrane and the Y-doped WO_3_ membrane, respectively. In the WO_3_ sensing membrane, there are two peaks at the binding energy values of 530.5 and 531.7 eV, which are the WO_3_ lattice and silicate, respectively. As the annealing temperature rose to 500 °C, the peak of the WO_3_ lattice showed the highest intensity. Conversely, the peak of silicate has the lowest intensity. This indicates that the WO_3_ sensing membrane annealed at 500 °C has the strongest bond strength. On the other hand, in the Y-doped WO_3_ sensing membrane, there are three peaks at the binding energy values of 529.6 [12], 530.4, and 531.7 eV assigned the Y_2_O_3_ lattice, WO_3_ lattice, and silicate. It also can be seen that the intensity of the WO_3_ lattice enhanced when the annealing temperature rose. The Y-doped WO_3_ sensing film annealed at 400 °C shows the highest intensity of WO_3_ lattice and the lowest intensity of silicate. Furthermore, the peak of Y_2_O_3_ can also be seen in the XPS spectra, which means that when doping to form the Y_2_O_3_ lattice in the material, as the annealing temperature rose to 400 °C, the intensity has a slight rise. Corresponding to XRD analysis, it also can be seen that the peak of Y_2_O_3_ emerges.

**Figure 4:**
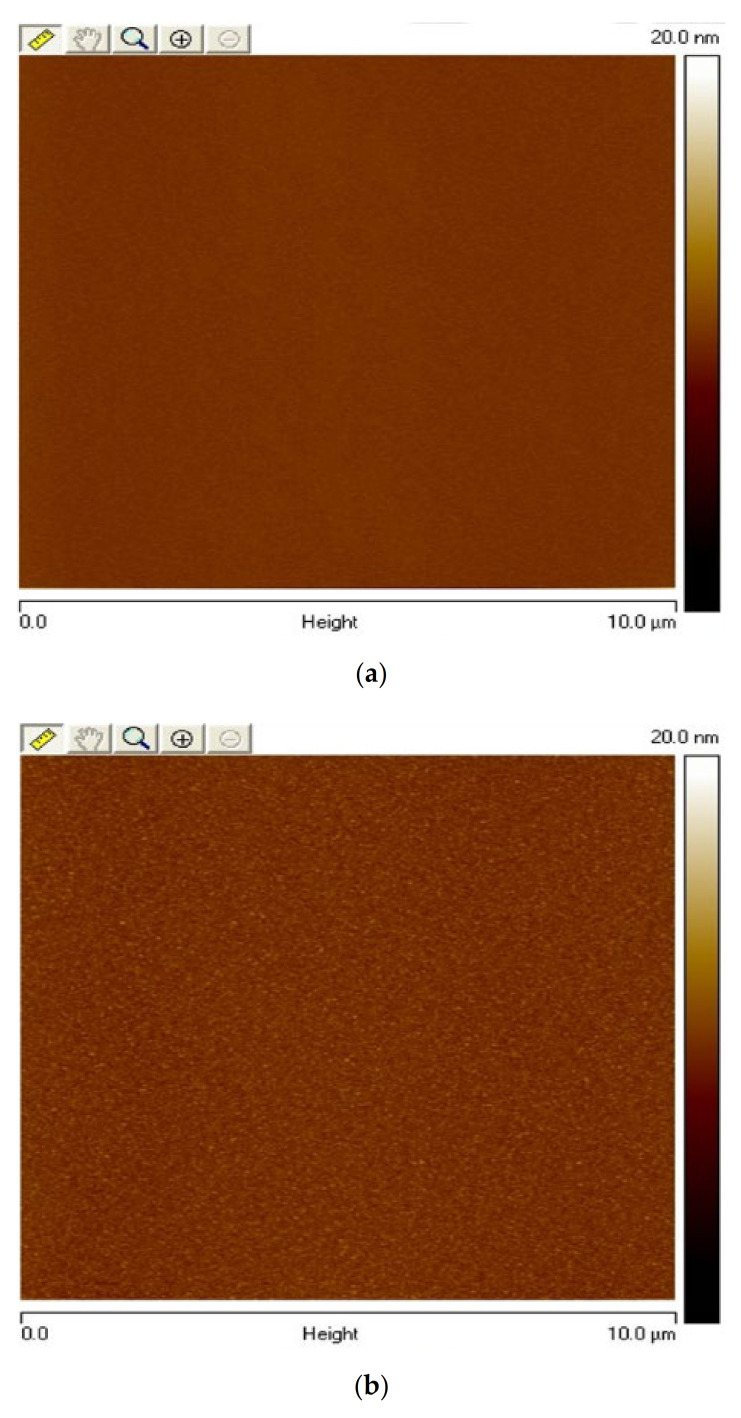

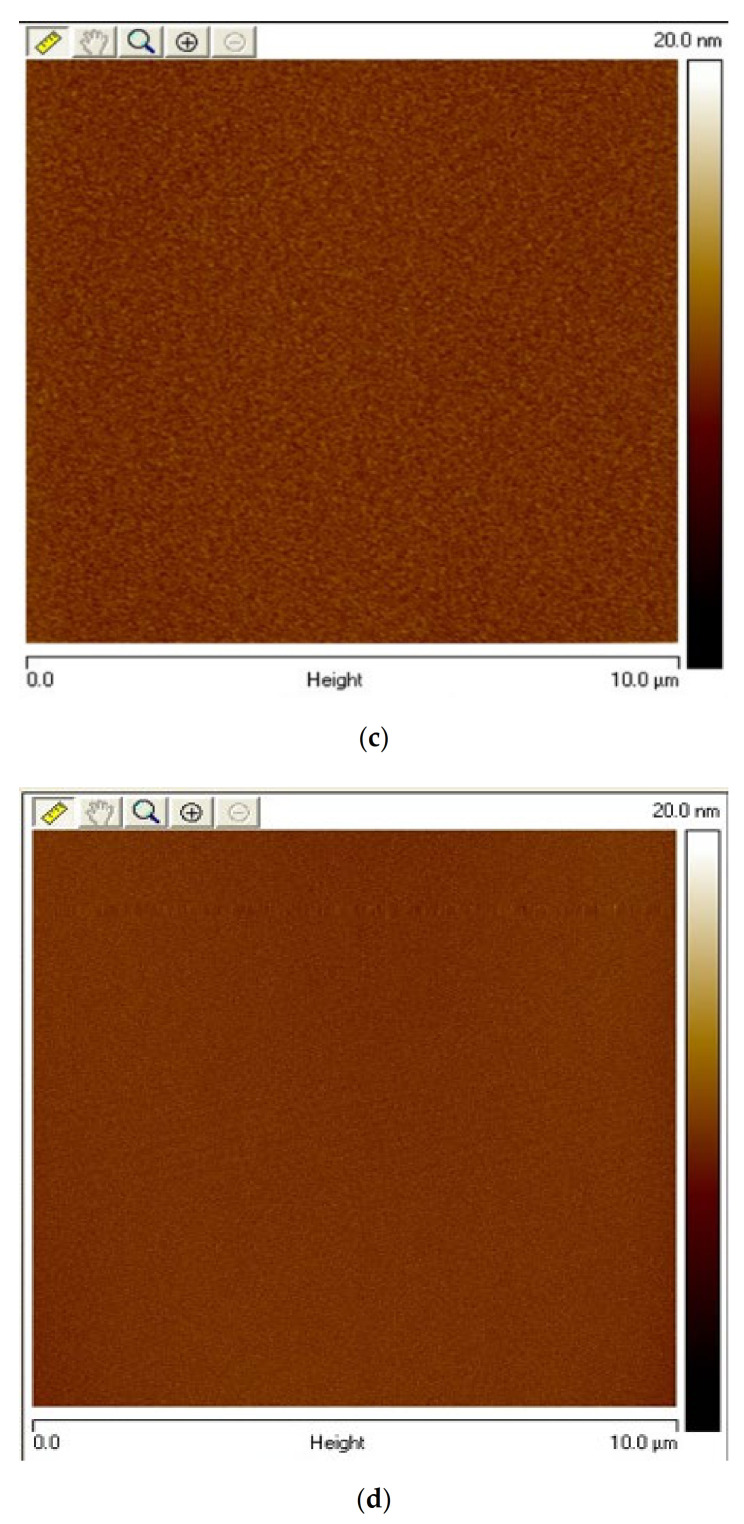

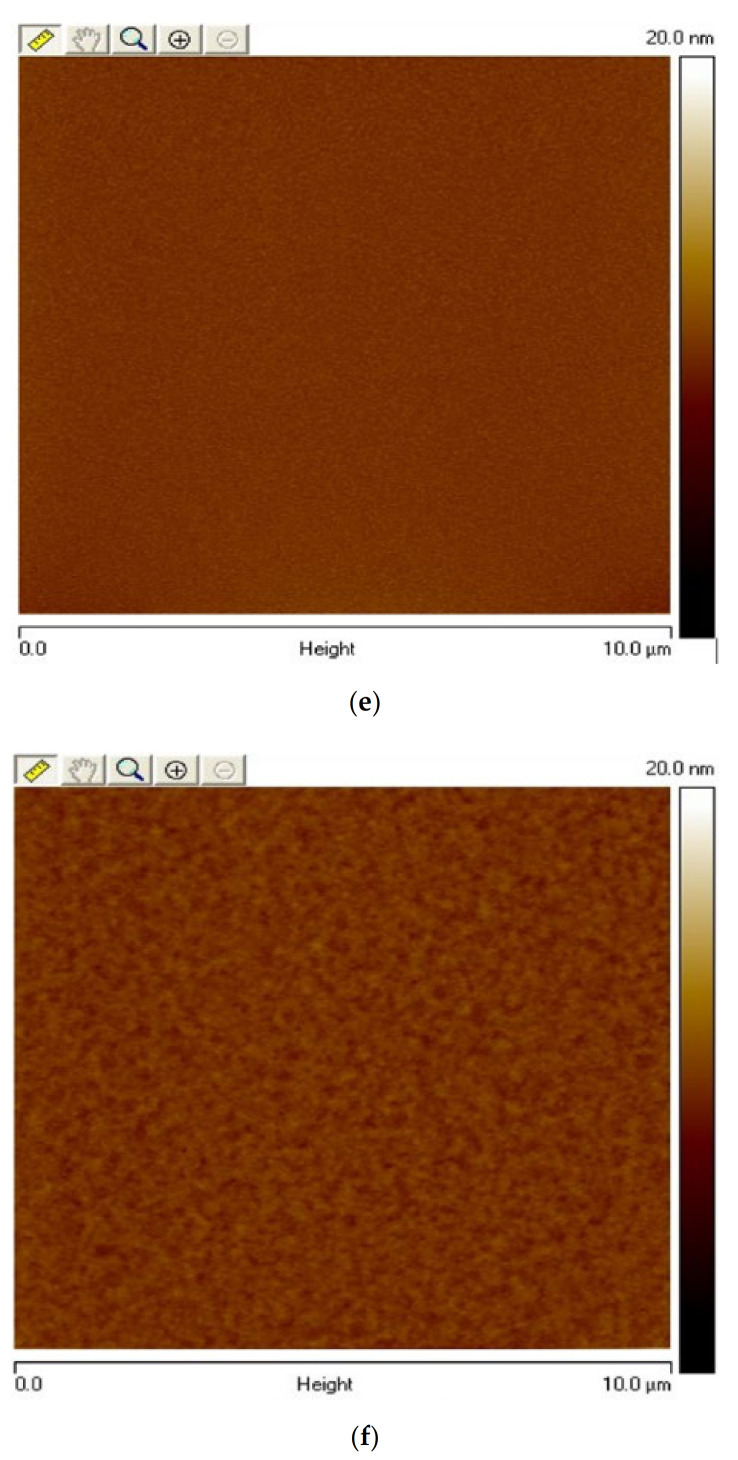

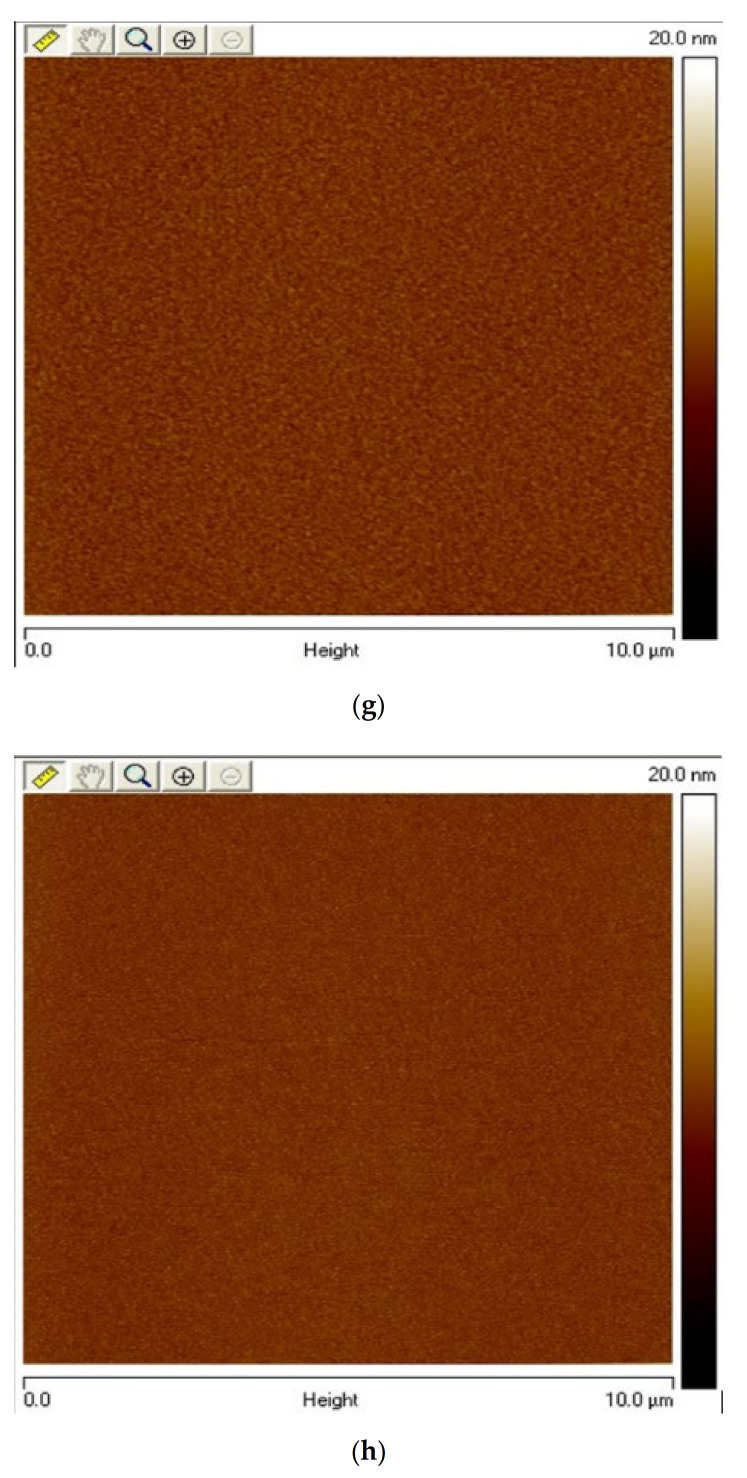
2D-AFM of WO_3_ film (**a**) as-dep RMS: 0.156 nm, (**b**) RTA 400 °C RMS: 0.354 nm, (**c**) RTA 500 °C RMS: 0.409 nm, and (**d**) RTA 600 °C RMS: 0.314 nm in O_2_ ambient for 30 s. 2D-AFM of Y-doped WO_3_ film (**e**) as-dep RMS: 0.29 nm, (**f**) RTA 400 °C RMS: 0.594 nm, (**g**) RTA 500 °C RMS: 0.474 nm, and (**h**) RTA 600 °C RMS: 0.464 nm in O_2_ ambient for 30 s.

Figure 4a–d show atomic force microscopy (AFM) 2D images of the WO_3_ sensing membrane with different RTA temperatures. Figure 4e–h show atomic force microscopy (AFM) 3D images of the Y-doped WO_3_ sensing membrane with different RTA temperatures. The root mean square (RMS) values of WO_3_ for as-deposited and samples annealed at 400, 500, and 600 °C are 0.156, 0.354, 0.409, and 0.314 nm, respectively. Furthermore, the root mean square (RMS) values of Y-doped WO_3_ for as-deposited and samples annealed at 400, 500, and 600 °C are 0.29, 0.594, 0.474, and 0.464 nm, respectively. Since Y^3+^ can enhance crystallization, the roughness of the Y-doped samples is greater than that of the undoped samples.

The operation of a sensing membrane can be realized as the metal oxide semiconductor capacitor in Metal-Oxide-Semiconductor Field-Effective Transistor (MOSFET) with an electrolyte and a reference electrode placed on the gate location. To assess the sensing behaviors electrolytes, C-V measurements are conducted. The connection between the substrate bias and the electrolyte concentration can be computed. Furthermore, the substrate bias voltage variation induced by the varying of electrolyte concentration can be explained by the site-binding model [33,34]. The shift of the flat band voltage is proportional to the electrolyte concentration as Equation (1):

(1) $$V_{\mathrm{FB}}=E_{\mathrm{Ref}}-\psi +\chi^{\mathrm{sol}}-\frac{\phi_{\mathrm{Si}}}{q}-\frac{Q_{\mathrm{ox}}-Q_{\mathrm{ss}}}{C_{\mathrm{ox}}}$$

$E_{\mathrm{Ref}}$ is the reference electrode potential and ψ is the junction potential difference. $\chi^{\mathrm{sol}}$ is the solution’s surface dipole potential.$\;\phi_{\mathrm{Si}}$ is the work function. ψ is correlated with the surface sites.

According to the AFM analysis, the surface of the Y-doped membrane sample annealed at 400 °C is the roughest. The β value is considered to be related to the sensitivity of the component and can be calculated by using the following equation:

(2) $$\psi =2.303\frac{\mathrm{kT}}{q}\frac{\beta }{\beta +1}\left({\mathrm{pH}}_{\mathrm{pzc}}-\mathrm{pH}\right)$$

Moreover, the hydrogen ion reaction with the membrane interface is illustrated in the site-binding model shown in Equation (2). The surface potential can be related to the membrane parameter β. k is Boltzmann’s, constant, q is the elementary charge, T is the temperature, and pH_pzc_ is the pH value with a zero charge on the surface. Furthermore, β is closely related to the density of surface hydroxyl groups, as shown in (3). N_s_ is the number of surface sites per unit surface area, and C_DL_ is the double layer capacitance, according to the Gouy-Chapman-Stern model.

(3) $$\beta =\frac{2q^{2}N_{s}\sqrt{K_{a}/K_{b}}}{{\mathrm{kTC}}_{d}}$$

The Y-doped sample annealed at 400 °C shows the increase of surface roughness and a higher number of surface sites, which caused better performance in sensitivity and linearity. According to the FESEM analysis, when the RTA temperature of the Y-doped sample rose to 400 °C, the membrane surface showed conspicuous grains. By examining XRD and XPS measurements, it can be explained that the yttrium could effectively combine with tungsten and yttrium atoms to form larger grains.

**Figure 5:**
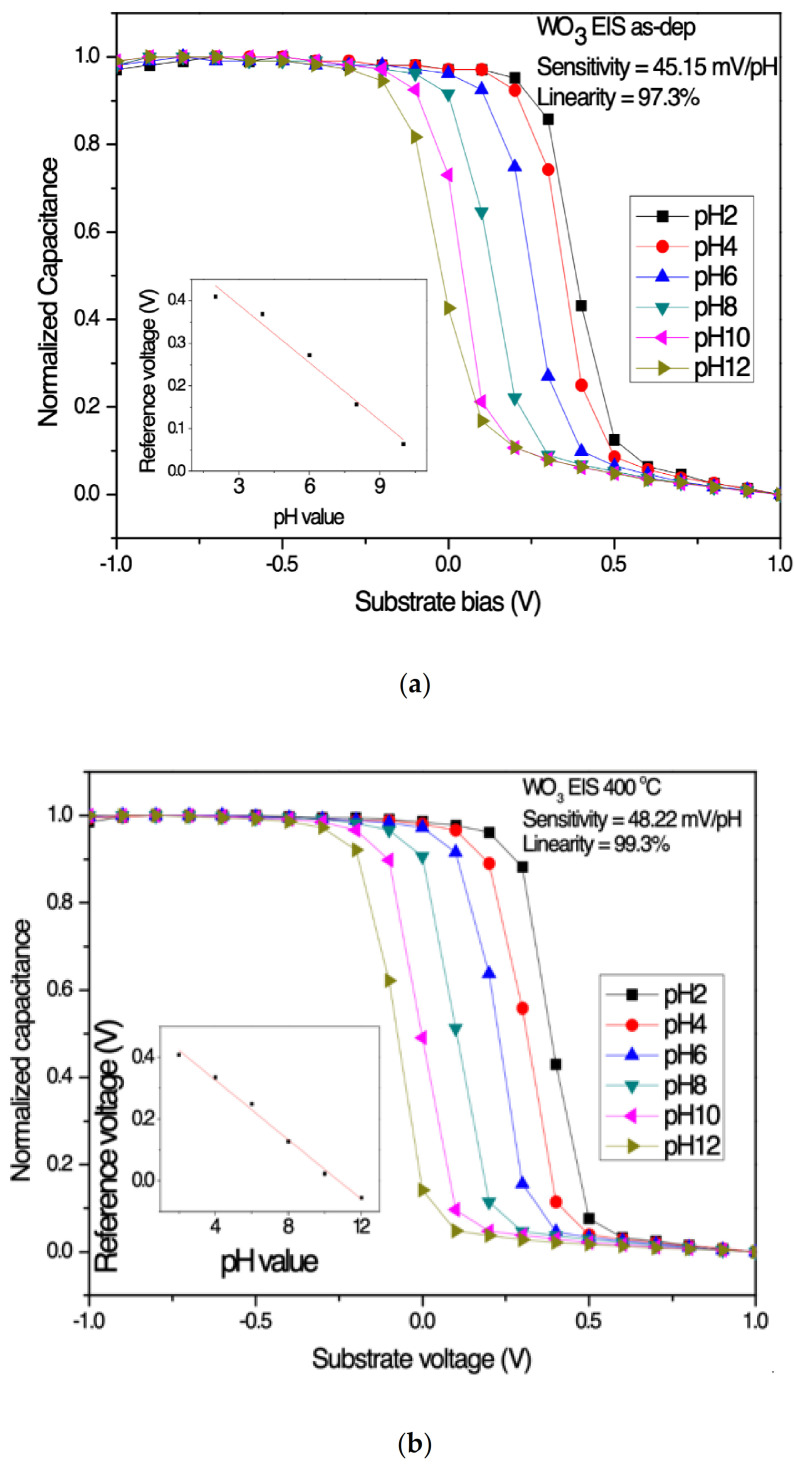

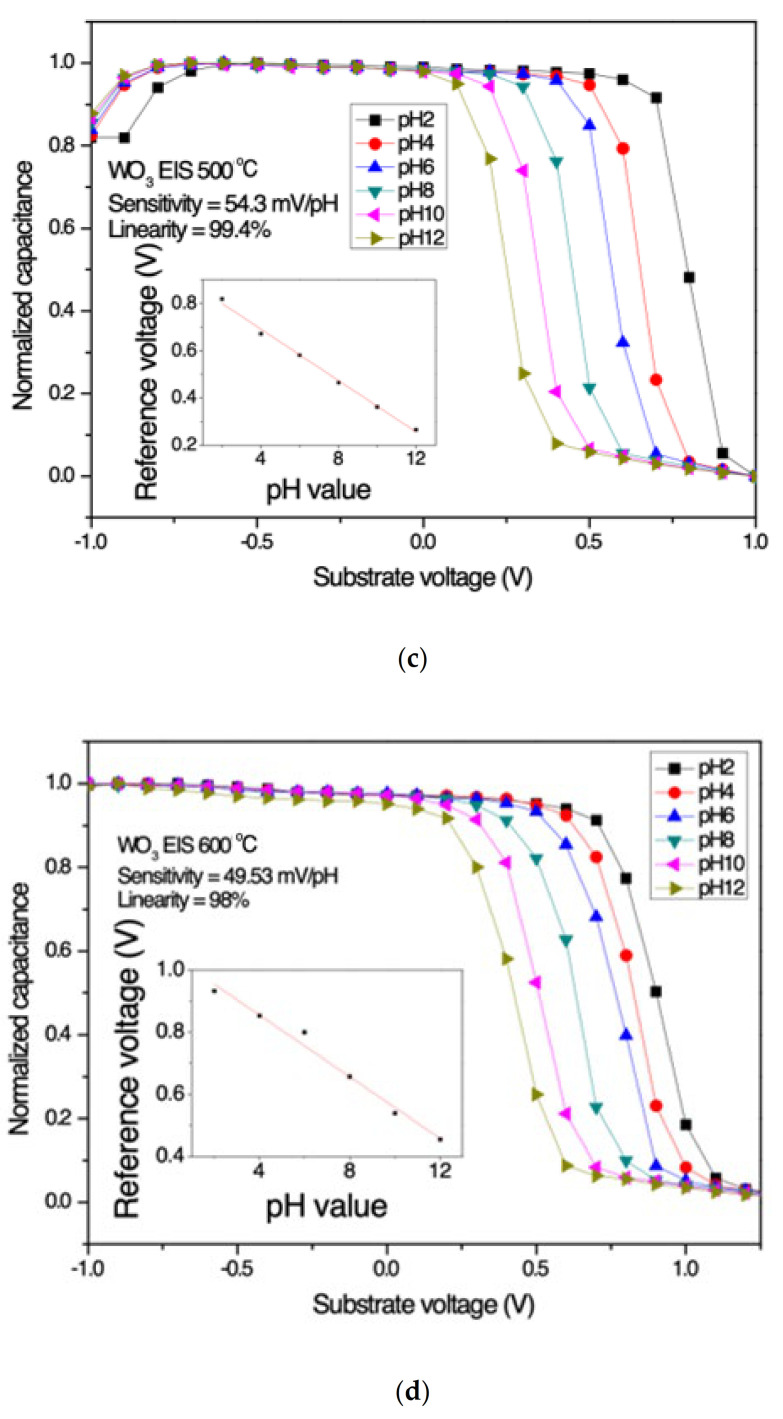

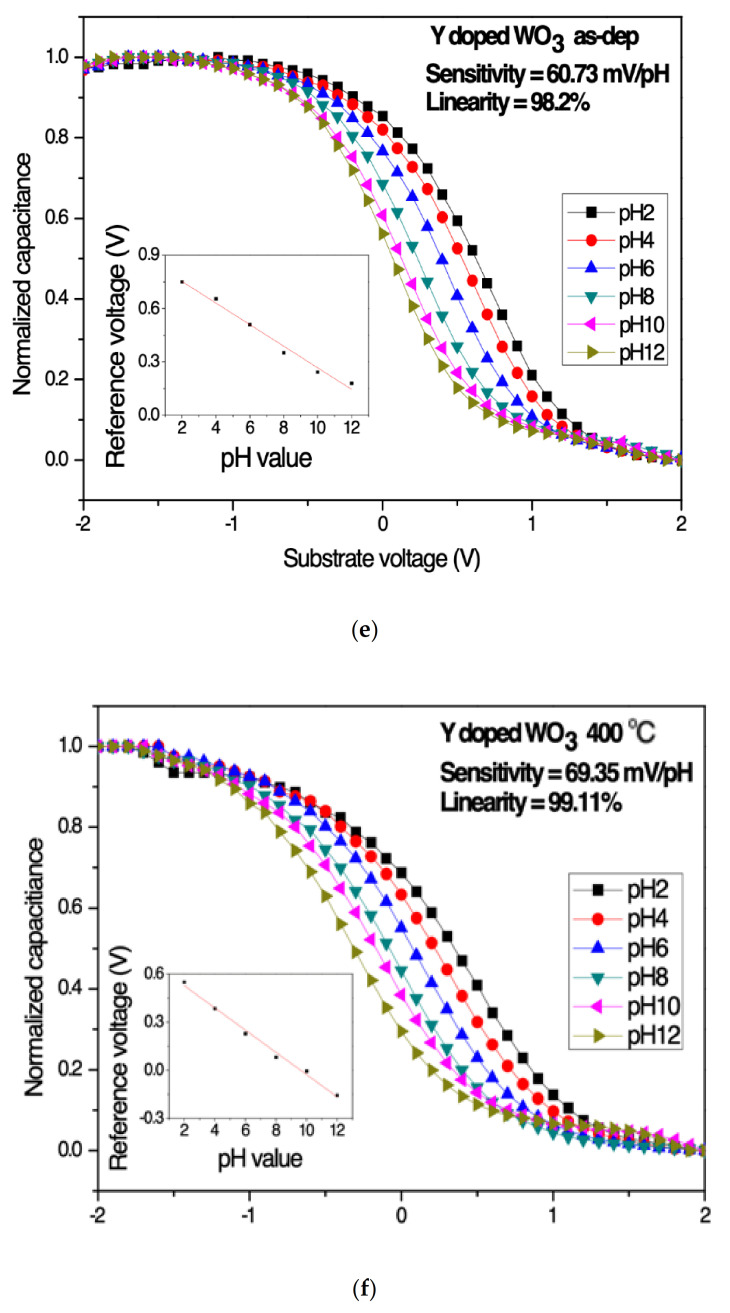

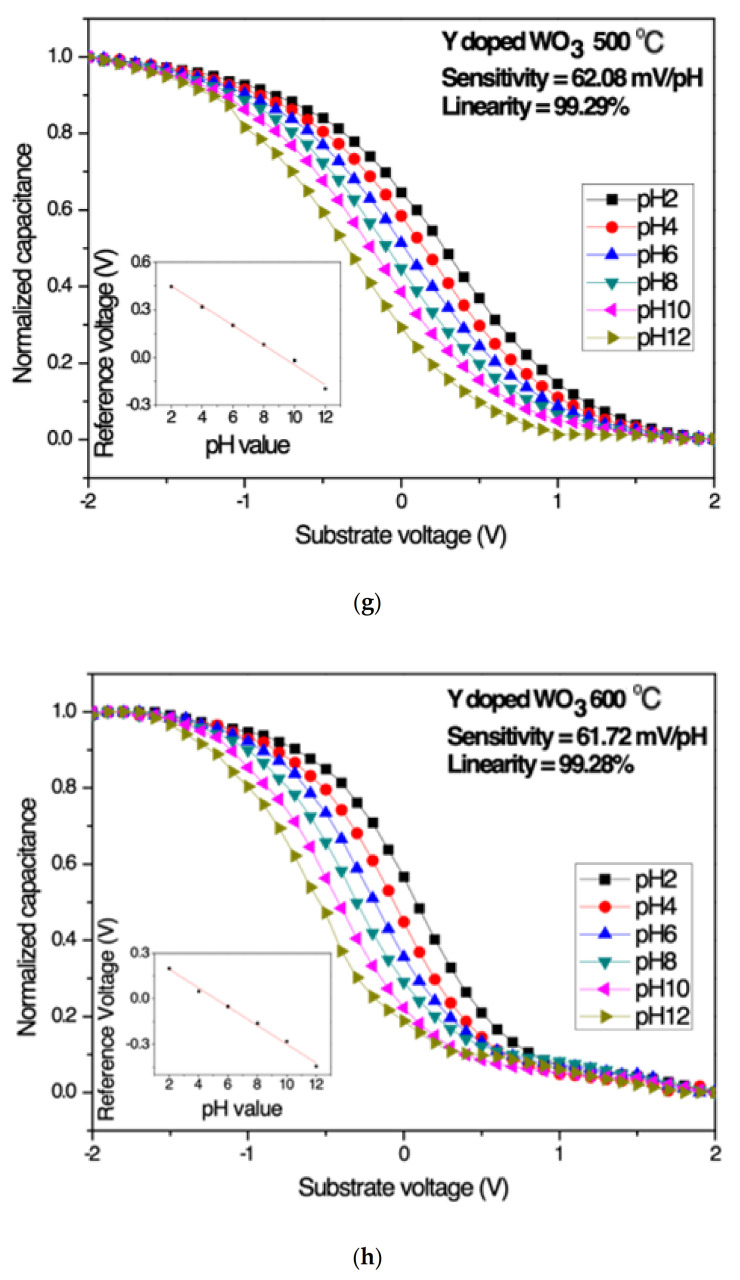
Sensitivity and linearity of the WO_3_ sensing membrane based on EIS structure annealing in O_2_ ambient with different temperatures (**a**) as-dep, (**b**) 400 °C, (**c**) 500 °C, (**d**) 600 °C. The sensitivity and linearity of the Y-doped WO_3_ sensing membrane based on EIS structure annealing in O_2_ ambient with different temperatures (**e**) as-deposited, (**f**) 400 °C, (**g**) 500 °C, and (**h**) 600 °C.

To measure the sensitivity and linearity of EIS capacitors, a Ketheley 2400 Source Meter is used to evaluate the C–V curves of the samples treated in various conditions. With 0.4 Cmax set as the reference capacitance, the sensitivity and linearity can be calculated by extracting the points of different pH values. All measurements are performed at room temperature. Figure 5a–h show C–V curves of WO_3_ and Y-doped WO_3_ sensing film annealed at different temperatures to evaluate the sensing performance. The sensitivity values of WO_3_ sensing film based on the EIS structure for the as-deposited, 400, 500, and 600 °C annealing are 45.15, 48.22, 54.3, and 49.53 mV/pH, respectively. The linearity values of the four samples for the as-deposited and the samples after annealing treatment are 97.3, 99.3, 99.4, and 98%, respectively. On the other hand, the sensitivity values of Y-doped WO_3_ sensing film based on EIS structure for the as-dep, the samples with 400, 500, and 600 °C annealing are 60.73, 69.35, 62.08, and 61.72 mV/pH, respectively. The linearity values of the four samples for as-deposited and post-annealing treatment are 98.2%, 99.11%, 99.29%, and 99.28%, respectively. The Y-doped WO_3_ sensing membrane showed better performance than the WO_3_ sensing membrane based on the above results. According to the physical analysis, it can be proved that the oxygen vacancy content obviously decreased in the Y-doped samples and the crystals formed. Therefore, the sensitivity and linearity are significantly improved. Compared with recent studies on pH sensing membranes, as shown in Table 1. Y doping and annealing can enhance effective electric field passing through WO_3_ dielectric and thereby improves the capacitance modulation as shown in Equation (1). Y-doped WO_3_ membranes with appropriate annealing have excellent sensing behaviors.

**Figure 6:**
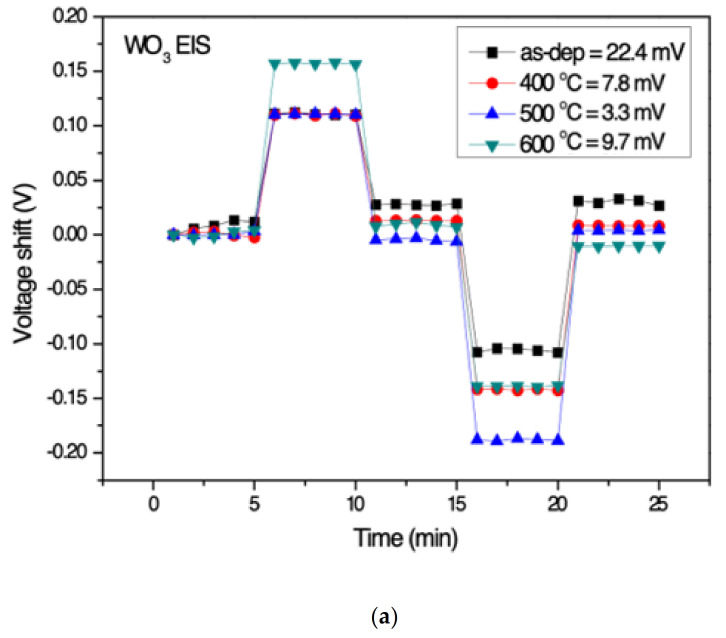

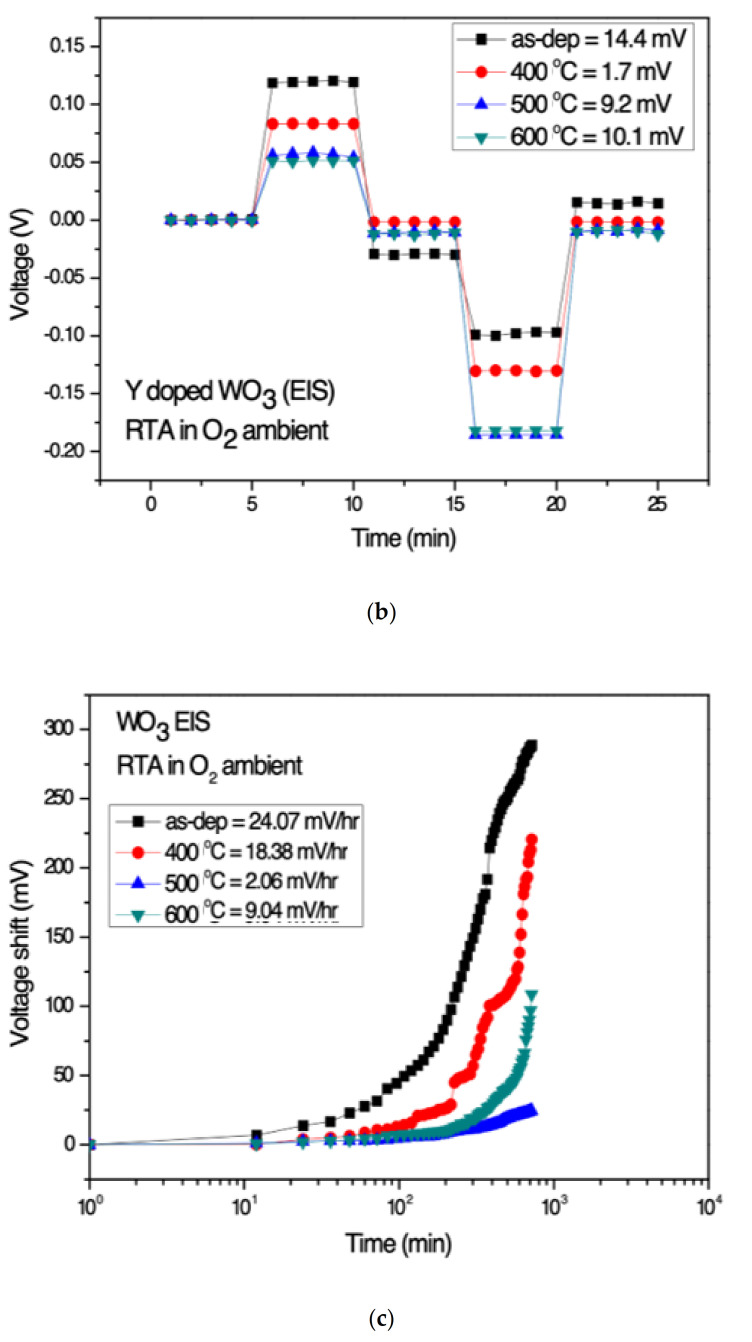

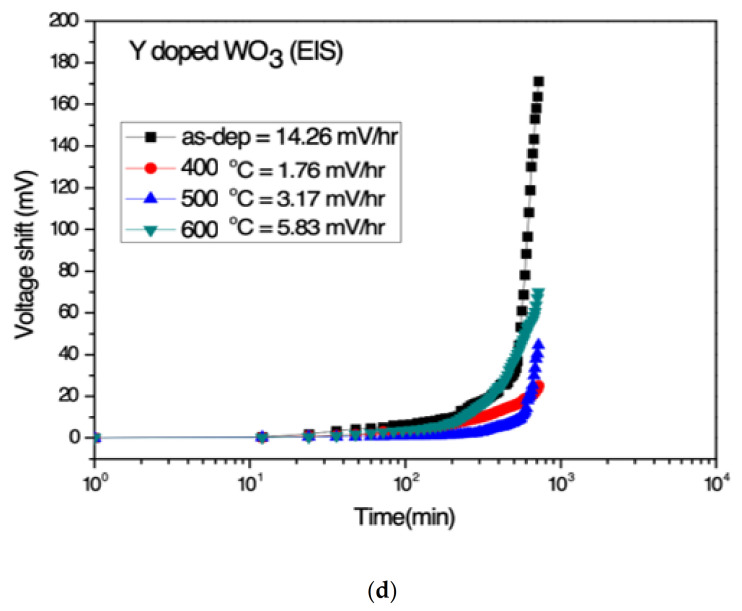
(**a**) Hysteresis voltage of WO_3_ sensing membrane based on EIS with various RTA temperatures in O_2_ ambient during the pH loop of 7→4→7→10→7. (**b**) Hysteresis voltage of Y-doped WO_3_ sensing membrane based on EIS with various RTA temperatures in O_2_ ambient during the pH loop of 7→4→7→10→7. (**c**) Drift voltage of WO_3_ sensing membrane based on EIS with various RTA temperatures in O_2_ ambient, then dipped in pH 7 buffer solution for 12 h. (**d**) Drift voltage of Y-doped WO_3_ sensing membrane based on EIS with various RTA temperatures in O_2_ ambient, then dipped in pH 7 buffer solution for 12 h.

To observe how the acid-based solution affected the property of devices, the samples are soaked in solution in the order of pH7-pH4-pH7-pH10-pH7. The setup is shown in Figure 1b. The hysteresis is the voltage measured for every minute. The value of the hysteresis voltages reflects the defect density on the film and the defect, affecting the gate voltage in the sensing membrane [39]. The hysteresis voltage is defined as the substrate voltage difference between the initial and terminal voltages measured in the pH loop. All the measurements are performed at room temperature. The hysteresis voltage of the WO_3_ samples and Y-doped WO_3_ samples based on EIS structure in different RTA temperatures are shown in Figure 6a,b. The hysteresis voltages of the WO_3_ for the as-dep sample and the samples with annealing temperatures of 400, 500, and 600 °C are 22.4, 7.8, 3.3, and 9.7 mV, respectively. On the other hand, the hysteresis voltage of Y-doped WO_3_ for the as-dep sample and the samples with annealing temperatures of 400, 500, and 600 °C are 14.4, 1.7, 9.2, and 10.1 mV, respectively. The Y-doped WO_3_ sensing film annealed at 400 °C has the lowest hysteresis voltage. Furthermore, compared with the samples without Y doping, a significant decrease in hysteresis voltage can be observed after Y doping, for Y doping suppresses the defects and inhibits the formation of oxygen vacancies. Therefore, the function of membrane capacitance can be ameliorated as shown in Equations (1)–(3). The results are consistent with XPS analysis and XRD patterns.

Moreover, the drift voltage is an indicator for analyzing the long-term stability of the device. We can use the model of gate voltage drift of pH-ISFET to describe the hopping or trap-limited transport mechanism and the realization of dispersive transport [40]. All the measurements are performed at room temperature with pH = 7. The configuration is shown in Figure 1b. The C–V curves of the drift effect of the WO_3_ sensing film and Y-doped WO_3_ are measured in a pH 7 buffer solution for 12 h, as shown in Figure 6c,d. The drift rate values of the WO_3_ samples for the as-deposited and the samples with RTA temperatures of 400, 500, and 600 °C are 24.07, 18.38, 2.06, and 9.04 mV/hr, respectively. Further, the drift rate values of the Y-doped WO_3_ samples for the as-deposited sample and the samples with RTA temperatures of 400, 500, and 600 °C are 14.26, 1.76, 3.71, and 5.83 mV/hr, respectively. Results indicate that Y doping and annealing can eliminate the trapping states in membrane capacitance as shown in Equations (1)–(3) enhance device reliability.

## 4. Conclusions

In this study, WO_3_ and Y-doped WO_3_ sensing membranes with various annealing conditions are fabricated in EIS structures. Results show that high sensitivity of Y-doped WO_3_ sensing membranes can be achieved. To gain insight into the improvements, multiple material characterizations are performed. Results indicate that a membrane annealed at an appropriate temperature exhibits higher sensitivity, higher linearity, lower hysteresis voltage, and lower drift rate than all other samples. Based on multiple material analyses, Y doping can enhance crystallization and chemical bindings because of the formation of Y_2_O_3_ and suppression of oxygen vacancies. Y-doped WO_3_ membranes show promise for future biomedical applications due to their stable response, compact size, and good sensing behaviors.

## Figures and Tables

**Table 1:** Recent state-of-the-art EIS pH sensors.

NO.	Year	Author	Sensing Material	Sensitivity	Linearity	Reference
1	2020	Chen et al.	APTES/SiO_2_	61.8 mv/pH	99%	[35]
2	2021	Zina Fredj et al.	HfO_2_	54.5 mv/pH	99.66%	[36]
3	2021	Pan et al.	LaTixOy (LTO)	68.17 mv/pH	99.96%	[37]
4	2022	Kao et al.	Sb_2_O_3_	60.17 mv/pH	99.06%	[38]
5	This Work	Kao et al.	WO_3_	69.35 mv/pH	99.29%	This Work
